# Derivation of species-specific hybridization-like knowledge out of cross-species hybridization results

**DOI:** 10.1186/1471-2164-7-110

**Published:** 2006-05-08

**Authors:** Carmiya Bar-Or, Meira Bar-Eyal, Tali Z Gal, Yoram Kapulnik, Henryk Czosnek, Hinanit Koltai

**Affiliations:** 1 Department of Ornamental Horticulture, ARO Volcani Center, Bet Dagan, Israel; 2 Department of Nematology, ARO Volcani Center, Bet Dagan, Israel; 3 Depatment of Agronomy & Natural Resources, ARO Volcani Center, Bet Dagan, Israel; 4 The Robert H. Smith Institute of Plant Sciences and Genetics in Agriculture, Faculty of Agriculture of the Hebrew University of Jerusalem, Rehovot, Israel

## Abstract

**Background:**

One of the approaches for conducting genomics research in organisms without extant microarray platforms is to profile their expression patterns by using Cross-Species Hybridization (CSH). Several different studies using spotted microarray and CSH produced contradicting conclusions in the ability of CSH to reflect biological processes described by species-specific hybridization (SSH).

**Results:**

We used a tomato-spotted cDNA microarray to examine the ability of CSH to reflect SSH data. Potato RNA was hybridized to spotted cDNA tomato and potato microarrays to generate CSH and SSH data, respectively. Difficulties arose in obtaining transcriptomic data from CSH that reflected those obtained from SSH. Nevertheless, once the data was filtered for those corresponding to matching probe sets, by restricting proper cutoffs of probe homology, the CSH transcriptome data showed improved reflection of those of the SSH.

**Conclusions:**

This study evaluated the relative performance of CSH compared to SSH, and proposes methods to ensure that CSH closely reflects the biological process analyzed by SSH.

## Background

DNA microarrays enable researchers to conduct large-scale quantitative experiments on gene expression, which can elucidate the mechanisms and the prediction of biological processes, the assignment of functions to previously un-annotated genes, the grouping of genes into functional pathways, and the prediction of the activities of new compounds [reviewed in [1]].

One approach to microarray-based genomic research is to use cross-species hybridization (CSH) by hybridizing RNA of the studied organism to a microarray chip which contains transcripts of genes of a closely related species. CSH may be used in such cases when a microarray of the studied organism is unavailable (e.g. [2-6]). This is true for many organisms for which extensive cDNA libraries are not available (e.g. [7]). CSH is also used for comparative genomics, where transcripts from closely related species are hybridized to a cDNA microarray derived from one of them, for revealing evolutionary conserved mechanisms and pathways of expression control [8-12].

Different microarray platform types have been used for CSH. Affymetrix oligo-micorarrays were used for CSH in several studies such as [13-18]. Some encountered difficulties in obtaining relevant biological knowledge from CSH (e.g. [13,15,18]). However, once sub-probe sets were created and used for data analysis, a marked increase in the ability to extract valid expression profiling from CSH occurred (e.g. [15,17]). Other CSH studies used custom oligos arrays (e.g. [19]). Once these were compared to affymetrix-CSH studies, it was suggested that longer oligos produce better results for CSH [19]. The longest probe microarrays available are the cDNA microarrays; these are assumed to be more suitable for CSH since cDNA probes may be sufficiently large so that small interspecies differences in nucleotide sequences might not affect the analytical results [4,8,20]. Indeed it seems that biologically meaningful information may be obtained from CSH over cDNA microarrays [2,3,5,6,10,11,21-25]. However, some CSH studies have demonstrated spurious results for CSH performed on cDNA arrays, even for closely related species [8,9].

The use of CSH presupposes the ability to analyze the gene expression profiles, so that biologically meaningful knowledge can be obtained. This paradigm was examined in several studies. Renn et al. [3] quantified gene expression profiles of closely related species using CSH. Their results suggested similar profiles for closely related taxa (that diverged >65 million years ago; MYA), but less similar profiles for distantly related species (that diverged ~200 MYA). By performing co-hybridization of RNA extracted from two species on a microarray platform that contained probes of both species, Gilad et al. [9] compared the experimental error between these two CSHs; where a third SSH experiment served as a reference. A marked effect of sequence mismatches on hybridization signal, even between organisms that are only ~1% diverged was demonstrated, indicating that even in this case CSH results might be biased.

To the best of our knowledge, our present study uses for the first time RNA samples from the same species, for both cross-species (heterologous) and species-specific (homologous) hybridizations. Thus, a direct comparison between results generated by CSH and SSH was feasible, and determination of the quality of CSH compared to SSH was determined.

## Results and discussion

To examine whether CSH can produce data that reflects SSH data, we used the same RNA samples for both CSH and SSH microarray experiments. RNA samples were extracted from nematode-infected and non-infected (control) potato plants, sampled at two time-points (5 and 10 days – both represent early time-points of nematode infection). These RNA samples (i.e. test samples) were co-hybridized with a reference sample by CSH to a tomato microarray and by SSH to a potato microarray. The reference sample was pooled from all 8 samples (2 time-points of infected or non-infected plants; 2 biological replicates were performed). The CSHs were designated as 'PT' for potato samples on tomato microarrays; the SSHs were designated as 'PP' for potato samples on potato microarrays. The data discussed in this publication including detailed description of the potato and the tomato microarray platforms and all of the PP and PT expression data, have been deposited in NCBI Gene Expression Omnibus (GEO; [26]) and are accessible through GEO Series accession number GSE3584.

### Microarray representation of species genes

To assess whether the tomato microarray may be used for representation of a biological process that occurs in potato, we determined the extent to which the tomato microarray represents known potato genes. On the one hand, ESTs, which are the only sequences available for the tomato and the potato microarray printed clones, may not support determination of gene representation; their short size may inhibit accurate identification of transcripts (i.e. lack of overlapping between them may not necessarily indicate different genes). On the other hand, the complete sequences of the microarray printed clones – although may be the ultimate sequence class for comparisons – are not available for the tomato and potato arrays. This might also be the case for other microarrays of spotted clones.

Therefore, we had to evaluate the complete clone sequences by using their assigned contigs (i.e. unigenes), and use unigenes for determination of tomato and potato microarrays representation of potato known genes. For this purpose, sequences of unigenes that were represented by the microarray platforms were used to blast against sequences of a potato unigene database (i.e. TIGR consensus build of potato, version 10.0). Sequences that matched with an E-value less than e-05 were considered homologous. Using this level of homology matching, the tomato microarray represents 29% (6,147 out of 21,063 potato TCs) of the potato known genes. In comparison, the potato microarray represents 51.5% (10,860 out of 21,063 potato TCs) of the potato known genes. On the tomato microarray, 83% (6,147 out of 7367) of the tomato genes were homologous to potato known genes, compared to 100% (10,860 out of 10,860) of the potato microarray genes that were found homologous to potato known genes (Additional file 1).

It should be noted that until a genomic database is complete, these representation levels may change. Species representation level may be affected by the dynamics of a unigene database built. That is because unigenes may be split and re-merge until a unigene final form is attained; they may reflect splice variants [27,28] and some of them may be incorrect [29]. Thus, once unigenes are chosen for determination of microarray representation, the completeness of a unigene database in terms of both the percentage of the genes that are already represented by unigenes in the database and their quality (i.e. how accurately they represent a real gene) should be considered. In addition, the origin of the libraries in terms of plant organ or developmental stage, used for microarray construction or gene database may also affect the representation level, as may be the case in this tomato-potato comparison.

Notably, cross-species representation levels may not necessarily be a proof of representation quality. Approximately 16% (992 of 6,147) of the potato unigenes registered more than one hit during blast between the tomato microarray unigenes and the potato unigene database. This may have been a result of sequence mismatches due to cross-species sequence divergence. Alternatively, multiple hits may result from genes being members of gene families (which may also be the case for SSH). Multiple hits might lead to a reduction in the accuracy of the reflection of the biological process, since 2 or more different transcripts might hybridize to the same spot on the array.

In conclusion, taking into consideration that the tomato microarray is a cross-species platform to potato, and contains less spotted clones than the potato microarray (9,140 and 15,254 spotted clones, respectively), the 6,147 potato unigenes represented by the tomato microarray (compared to the 10,860 potato unigenes represented by the potato microarray) suggest that the tomato microarray may be used in the present study for potato gene expression profiling.

In addition, Renn et al. [3] performed a phylogenetic-based evaluation of microarray representation of the genomes, and similar expression patterns were observed for organisms that diverged up-to 65 MYA and even to some extent for organisms that diverged up to 200 MYA. Based on non-synonymous and synonymous distances, it has been suggested that potato and tomato diverged about 17 MYA [30,31]. Taken together, the study of Renn et al. [3] and the phylogenetic assessment of the potato-tomato divergence support the above premise, i.e. that potato-tomato CSH may be used for obtaining biologically meaningful results.

### Comparison of CSH and SSH transcriptomic (whole chip) results

To determine the extent to which CSH may reflect SSH transcriptomic results, we have performed various analyses for each of PT or PP whole chip expression data, and compared their results. We first, determined the average correlation coefficient of the reference sample signal between all pairs of hybridizations within PT or PP experiments. For this purpose we used present and marginal calls separately for PT or PP (14,868 and 9,140 clones of the PP and PT experiments, respectively, had present or marginal calls). Reference sample signal correlation coefficient of 0.88 (SD = 0.036, N = 28) or 0.89 (SD = 0.02, N = 28) was determined for PP or PT experiments, respectively. The similarity between the correlation coefficients suggests hybridization quality consistency for the same RNA sample for CSH, compared to SSH. This similarity implies repeatability of the CSH between the RNA and the microarray probes, despite low homology matches of a transcript to a spot, or where more than one transcript hybridizes to one spot. Although these 2 cases may also be present in SSH (e.g. different members of a gene family may hybridize to one spot in SSH), it is likely that they are more abundant in CSH.

**Figure 1:**
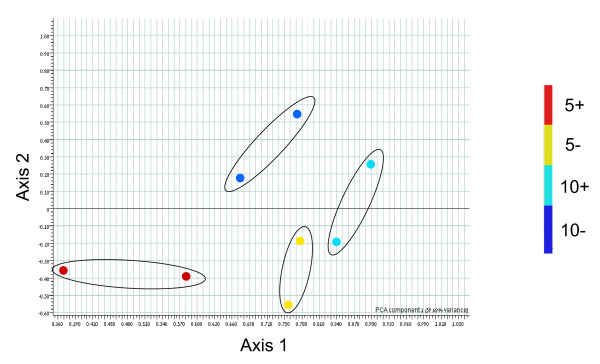
**Principal component analysis (PCA) of the expression matrix of the species-specific hybridization experiment**. PCA of 14,868 genes, across 8 microarray hybridizations of potato RNA to potato microarray (PP). Axes 1 and 2 account for 59.7 and 15.6 % of the variation, respectively. The hybridization datasets are labeled according to treatments; '+' or '-' designate infected or non-infected tissues, respectively; '10' or '5' designate 10 or 5 days of nematode infection. Biological replicates are circled.

Second, Principal Component Analysis (PCA) results were obtained as another parameter for the resemblance between CSH and SSH data. PCA was determined based on test-sample:reference-sample ratios for each of the PT or PP datasets. Two components (with cumulative variance of 73%; biological replicates matched to some extent, as indicated by their distance in the PC space) were detected by PCA for the PP transcriptomic data (Fig 1). Only one component was found for the PT transcriptomic data (not shown). This may suggest that despite the similarity in the quality of hybridization consistency (suggested by the similar reference-correlation coefficients), CSH may not accurately reflect SSH PCA results.

Third, the gene expression range was determined from the lower 0.1 to the upper 99.9 percentiles for all 8 test-sample:reference-sample ratios, and PP compared with PT. A clear reduction by a factor of 0.76 (on average, SD = 0.24, N = 8) in the ratio was observed for PT compared to PP. This is in agreement with other CSH studies. Renn et al. [3] have found a clear reduction in CSH signal, which was proportional to the phylogenetic distance of the 2 CSH-involved species. Adjaye et al. [8] pointed to differences in expression levels between SSH of human/human and CSH of bovine/human. Reduction in the signal observed for CSH, may be due to lack of specific RNA hybridization, resulting from reduced homology between potato and tomato, or from cases where more than one potato transcript hybridized to a single tomato clone.

**Figure 2:**
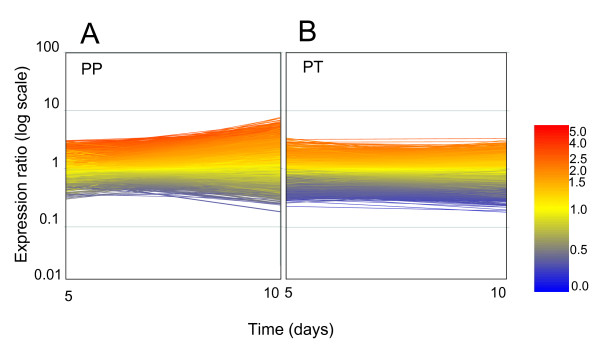
**Gene expression profiles of cross-species and species-specific hybridization data**. Gene expression profiles resulted from hybridizations of potato plants RNA to **(A) **a TIGR potato microarray (PP experiment), and **(B) **CGEP tomato microarray (PT experiment). Gene expression profiles are presented for (nematode-infected tissues-) test-sample:reference-sample (mixed samples) ratio, averaged over biological replicates, of 14,868 and 9,140 clones of PP and PT experiments, respectively. The transcriptional profile is drawn between 5 and 10 d of nematode infection. The color scale represents gene expression ratios.

Consequently, when gene expression profile was plotted between 5 and 10 d, for the (infected-) test-sample:reference-sample, for both PP and PT (Fig 2), the PP data exhibited amplification of gene expression from 5 to 10 d (Fig 2a). Similar amplification of gene expression from 5 to 10 d of nematode infection was also suggested in our previous study where tomato RNA was hybridized to a tomato microarray (i.e. tomato SSH experiments; [32]). For both the previous tomato and current potato SSH data, this may reflect a biological process common to the 2 closely related species. However, examination of the corresponding CSH data of PT resulted in masking of this biological process; no amplification of gene expression was evident from 5 to 10 d of nematode infection (Fig 2b).

Lastly, we wanted to examine whether the observed inconsistent reduction may be overcome once 2 hybridizations, which are a pair of a paired-observations, were compared. A pair of a paired-observation in our system is nematode-infected hybridization and the corresponding non-infected hybridization (e.g. '5a+' and '5a-'). The fold change for each pair was determined and averaged over biological replicates (i.e. over '5a' and '5b' and over '10a' and '10b'). This value served for identification of differentially regulated (i.e. >2 fold or <0.5 fold) genes for each time-point. For PP whole chip expression, data resulted in 591 and 790 differentially regulated genes at 5 and 10 d, respectively; only 80 and 52 differentially regulated genes were identified at 5 and 10 d in the PT whole chip data. Notably, the relatively low numbers of PT-differentially regulated genes compared to those of PP may have occurred, despite taking a liberal cutoff (2 fold). Therefore, the inconsistent reduction in signal intensity may not be overcome in our experiments by pair-wise fold determination.

In conclusion, the reduction in the CSH hybridization results (the ratio between sample and reference), along with its inconsistency, compared to the SSH hybridization result, may bias gene expression profiling of a biological process. This was manifested in the inability to identify PCA components, in aberrant gene expression profiling on a transcriptomic scale and in a decrease in the number of differentially regulated genes. Thus, our results suggest that the overall CSH data does not support the use of a whole chip (transcriptomic scale) analysis to provide a SSH comparative data profiles.

### Construction of tomato microarray – potato microarray matched probe sets

Inconsistency between hybridization results obtained from different platforms may be merely due to the fact that different platforms were used [33], whereas consistency between different platforms could be increased once a subset of data corresponding to cross-platform matched probe sets are used [27]. Therefore, to facilitate cross-platform and, hence CSH-SSH comparison, we created matched probe sets composed of probes from one platform (tomato microarray) that are homologous with probes from the other platform (potato microarray).

Mecham et al. [27] suggested that for comparison of 2 platforms, the best sequences to compare are overlapping probe-sequences that are spotted on the microarray (clone sequences in our system). This may avoid the representation of, among others, splice variants or incorrect assembled unigenes ([27] and references therein). Due to the unavailability of the complete clone sequences in our system, unigenes served as our choice for platform comparison (discussed in *Microarray representation of species genes*). Note that since overlapping of sequences may be essential once cross-platform comparison is considered [27], a possible drawback of using unigenes for this purpose is the possibility that 2 clones, being different parts of a corresponding homologous pair, do not overlap. According to [27], this might lead to inconsistency between the two platforms data, which correspond to these homologous pair probe sets. In summary, using unigenes for inter-platform comparison is inevitable despite the above described drawbacks due to the lack of complete clone sequences.

To create a unigene-based matched tomato-potato microarray probe sets, sequences of 7,637 tomato SGN unigenes (represented by 9,140 tomato microarray printed clones) served as queries to blast against 10,860 potato TIGR unigenes (represented by 15,264 potato microarray printed clones). BLAST resulted in 13,052 tomato SGN unigene – potato TIGR unigene homologous (E value ≤ e-1) pairs. This liberal match cut-off was used to gain a large range of E-values to be examined at later stages. These unigene homologous pairs were found to be representatives of 17,325 clone homologous pairs (i.e. tomato clone – potato clone pairs); each clone generated more than one pair, therefore, the number of homologous pairs is greater than that of the microarray spotted clones. The clone pairs served as probes for a System Expression Matrix (SEM) that was created by merging PP and PT probe corresponding data. SEM was utilized for further CSH and SSH analyses (see below).

### SEM probe pair homology optimization to gain maximal match of PP and PT data

A measure of reflection of the SSH data by the CSH data may be having all the biological replicates at 5 and 10 d (i.e. the infected:non-infected ratios of 5a, 5b, 10a and 10b) clustered for the PT and PP data. Therefore, according to this measure, SEM data was filtered according to tomato microarray – potato microarray probe homology (i.e. E-value or bit-score) to create subsets of SEM. Clustering results of these subsets were examined. E-value is a function of bit score, but weighs parameters such as unigene dataset size [34]; therefore, both E values and bit scores were used as parameters for sequence homology.

**Figure 3:**
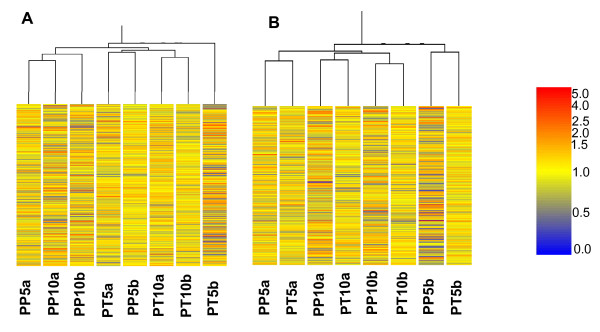
**Hierarchical clustering of cross-species and species-specific hybridization data corresponding to cross-species matched probe sets**. Hierarchical clustering of biological replicates by using Centered Pearson correlation, applied to (nematode-) infected:non-infected (potato plants) ratios of **(A) **The whole System Expression Matrix (SEM), comprising PP and PT data of 17,325 potato-tomato pairs with homology level of E-value ≤ e-1. **(B) **A subset of SEM, corresponding to 7,116 pairs with homology level of bit-scores ≥ 129 (or E-values ≤ e-26). The first letter of a condition name designates the RNA source ('P' for potato plants); the second letter indicates the microarray chip platform ('P' or 'T' for TIGR potato or CGEP tomato microarray chip, respectively); '10' or '5' designates days of nematode infection; 'a' or 'b' designates biological replicates. Color scale represents gene expression fold change.

**Figure 4:**
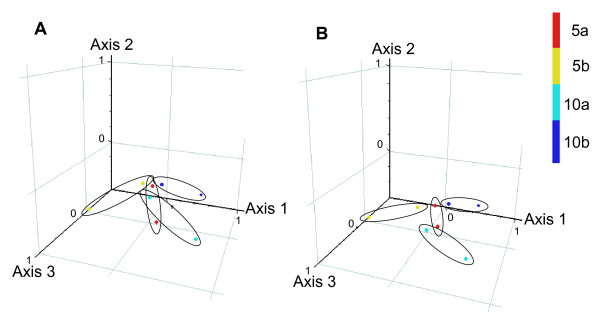
**Principal component analysis (PCA) of subsets of the System Expression Matrix (SEM) of cross-species and species-specific hybridization experiments**. PCA of (nematode-) infected:non-infected (potato plants) ratios of **(A) **The whole System Expression Matrix (SEM), corresponding to 17,325 potato-tomato clone pairs (E value ≤ e-1); **(B) **A subset of SEM, corresponding to 7,116 potato-tomato clone pairs (bit-scores ≥ 129 or E-values ≤ e-26). PCA was performed across two time-points (5 and 10 d of nematode infection) and two biological replicates (labeled as 'a' or 'b'), resulted from species-specific hybridization (SSH) of potato RNA to potato microarrays (denoted as PP) and from cross-species hybridization of potato RNA to tomato microarrays (denoted as PT). Axes 1, 2 and 3 account for **(A) **31.0, 21.3 and 13.8 %, **(B) **31.0, 23.0 and 14.6 % of the variation, respectively. Biological replicates are circled.

Hierarchical clustering of biological replicates of the unfiltered SEM data (infected:non-infected ratios) for each of the 17,325 homologous pairs resulted in no matches (Fig 3A). PCA of the same dataset resulted in an approximate grouping of the PT and PP biological replicates in a 3 principal component space (Fig 4A; Additional file 2). However, filtering of SEM data (infected:non-infected ratio) for higher bit scores improved the PT and the PP match (i.e., more biological replicates clustered over PP and PT). A perfect match between PT and PP (i.e. all biological replicates clustered over PP and PT) was observed for a subset of SEM which contained 7,116 homologous pairs with bit-scores higher than 129 (E-value ≤ e-26) (Fig 3B). PCA of this subset of SEM demonstrated improved grouping of the PT and PP biological replicates in a 3 principal component space (Fig 4B). Notably, the perfect match obtained for PT and PP was based only on optimization of the homology of sequences between the 2 examined species. This establishes the affect of species rather than any other affect such as that of dye, scanning, laboratory, etc. on the derivation of SSH-like results from CSH.

Lastly, filtering out the data of pairs that contained a tomato chimeric clone (i.e. clones that were found homologous to 2 unigenes, according to the manufacturer published data [35]; GEO [26] accession no.: GPL3034) resulted with 13,285 homologous pairs. For these, lower bit-score (105 compared to 129) and higher E-value (e-23 compared to e-26) thresholds lead to a perfect match between PP and PT. This "lowering of threshold" for a perfect match between CSH and SSH, once chimeric clones were filtered out, further strengthens the previous notion that the association between a clone (printed on the microarray chip) and its representative unigene may affect the interpretation of a CSH experiment results.

### Determination of the ability to detect differentially regulated genes with CSH

**Figure 5:**
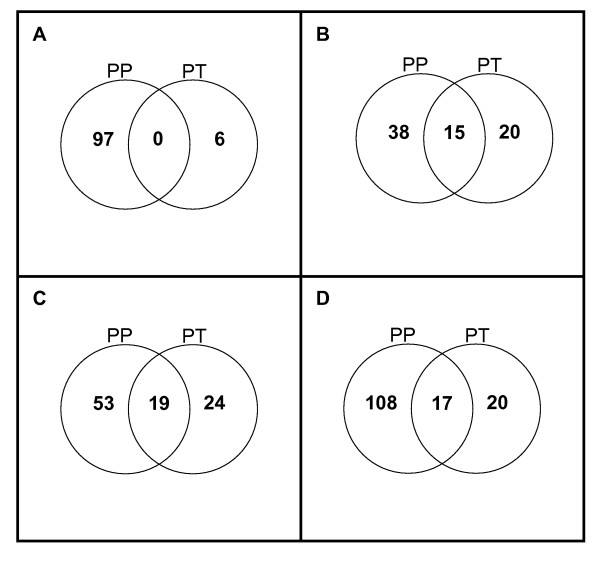
**Intersection of differentially regulated gene lists identified by cross-species and species-specific hybridizations**. Intersections performed between differentially regulated gene lists, identified by cross-species (PT) or species-specific (PP) hybridizations. Differentially regulated genes were identified between nematode infected and non-infected potato plants (based on infected:non-infected ratios). Intersections were performed for each time-point of the experiment (i.e. 5 or 10 d) and for each tendency of regulation (i.e. up or down). The PP and PT data corresponded to the sub SEM data filtered to include potato-tomato matched probe pairs with homology of bit-scores ≤ 129 (or E-values ≥ e-26). The intersection area presents the number of potato-tomato clone pairs that were differentially regulated for both PT and PP data. The first letter indicates the RNA source ('P' for potato plants); the second letter indicates the microarray chip platform ('P' or 'T' for TIGR potato or CGEP tomato microarray chip, respectively). (A) Down-regulated genes at 5 d; (B) up-regulated genes at 5 d; (C) down-regulated genes at 10 d; (D) up-regulated genes at 10 d.

Following probe-homology optimization of SEM data, we determined the extent to which may the SEM subset data that corresponded to bit-scores >129 (or E-values <e-26), support identification of differentially (>2 fold or <0.5 fold) regulated genes, identified in both the CSH and the SSH data. For this purpose, differentially regulated genes were identified based on the infected:non-infected ratio, averaged over 2 biological replicates, for each time-point and for each tendency of regulation (Additional file 3). Mutual differentially regulated genes (Additional file 4) comprised 43% or 16% (average values, N = 4, calculated based on the values in Fig 5) of the PT or the PP total number of differentially regulated genes. Thus, despite the generation of the SEM data and its probe homology optimization, a relatively small proportion of differentially regulated genes in our system was found mutual between the CSH and SSH results.

Of the PT data, 57% were false positives (i.e. differentially regulated genes identified for PT and not for PP data); whereas 84% were false negatives (i.e. differentially regulated genes identified for PP and not for PT data). A large number of false negatives may result from a reduction in the number of differentially regulated genes in CSH, compared to SSH. This was also indicated by other CSH studies (e.g. [3]). However, these differences between CSH and SSH observed in our system may have also resulted from other technical effect (such as differences in scanning protocols, dye labeling orientation, laboratories, etc.).

## Conclusions

In the present study, we have directly compared results generated by CSH with those generated by SSH to determine the quality of the CSH results and the steps that should be taken in order to extract valid results from CSH. The results show difficulties in inferring transcriptome data from CSH with that obtained from SSH. However, once the information has been filtered for those data corresponding to matching probe sets by restricting proper cut-offs of probe homology, the CSH data reflected more closely that of the SSH to an extent that was quantified by identification of differentially regulated genes.

Notably, all of the results that were generated in our PP-PT system may be specific to these particular platforms and to this particular experiment. A deduction from this system to other systems may not be straightforward. It is required that additional experiments will compare CSH to SSH using the same RNA samples (as in our system) in order to deduce general conclusions regarding derivation of SSH-like results from CSH data. Nevertheless, we suggest that this study may provide the reader with an approach to CSH. This may include guidelines for evaluating a CSH system, analyzing CSH data and assessing the quality of CSH results.

1. Although evaluation of the phylogenetic distance between the 2 CSH organisms (the one of the microarray and the one in study) may indicate on the CSH ability to reflect a biological process [3], once genomic data are available for the organism in study, we suggest that an evaluation of the cross-species microarray representation level of the studied organism may be performed by sequences comparison. An exact "formula" that would give a clear assessment for the level of microarray representation still needs to be developed to enable assessment of a CSH candidate platform.

2. Our data indicated that CSH whole chip results may not reflect SSH transcriptomic scale gene expression profiles. Thus, we suggest that the CSH transcriptomic data may not be trusted to reflect biological processes.

3. To facilitate extraction of SSH-like knowledge out of CSH, we have generated cross-platform (and hence cross-species) matched probe sets. We suggest that the creation of corresponding probe sets be performed for each and any CSH system, for which genomic data is available. Once considering the level of homology for cross-species comparison, the bit-scores (or E-values) should be optimized to the studied system. This may be easily performed in cases, where a species-specific platform exists, by maintaining a few RNA samples of the CSH experiment and hybridize them with the species-specific platform. This SSH supplemental experiment will allow a threshold determination for the specific CSH experiment. As for the rest of CSH systems, where a matched species-specific platform is not available, we suggest that the estimates presented here as a reference to start with, although these may be too strict; this particular experiment includes dissimilarities between CSH and SSH that may have been due to technical differences between the CSH and SSH systems. Finally, cross-species matched probe sets may be generated only for organisms with sufficient genomic data. As for other organisms, methods for extraction of biologically relevant knowledge from CSH need to be developed.

4. The generation of the cross-platform matched probe sets was based on unigene sequences. Since several factors such as genomic database information of the studied species may affect the degree of clone representation by unigenes, an ideal candidate for sequence comparison may be the spotted-clone sequences [27]. These should be fully sequenced to facilitate extraction of biologically relevant knowledge out of CSH.

## Methods

### Tomato and potato microarray description

Two cDNA microarrays, a tomato microarry and a potato microarray were used. The tomato cDNA microarray was developed and printed by the Center of Gene Expression Profiling (CGEP; Cornell University, Ithaca, NY); it contains 9,140 sequenced clones, selected at random from a number of different cDNA libraries derived from a range of tissues including leaf, root, fruit and flower [35]. The potato cDNA microarray version 3 was developed and printed by The Institute for Genomic Research (TIGR, Rockville, MD). The potato microarray contains 15,264 cDNA clones, selected from the potato stolon, root, microtuber, dormant tuber, germinating eye, healthy leaf, and phytophthora infestans-challenged libraries [36]. A description of the tomato and the potato microarrays is also available at GEO [26]; accession no. GPL3034 and GPL1902, respectively).

### Determination of potato gene representation level by the tomato and the potato microarrays

To determine the representation level of potato genes in the tomato microarray, its unigene sequences served as queries for a local BLAST search against a potato unigene database. IDs of the tomato microarray represented unigene were retrieved from the manufacturer [35]. Sequences of these unigenes were retrieved from the Solanaceae Genomics Network (SGN; [37]). Sequences of potato unigenes were retrieved from TIGR potato TC database (release 10.0). For the creation of a potato database index file, we used the NCBI FORMATDB utility. NCBI local BLASTN matched homologous (E-value ≤ e-05) tomato microarray – potato unigenes, according to their sequences. The total number of the tomato microarray represented potato TCs was divided by the total number of potato TCs.

As a reference to species-specific genes representation, we evaluated the potato genes representation level by the potato microarray. This was done by dividing the number of the potato microarray represented potato TCs by the total number of potato TCs.

### RNA source

RNA was extracted from nematode-infected and non-infected (control) potato plants. Two time-points of nematode infection were examined, 5 and 10 d. For each time point, two paired observations were applied. All paired observations were taken at the same greenhouse, but at different times. Each paired observation consisted of 2 groups of 6 plants, grown on adjacent plates. Six plants were nematode-inoculated and the other 6 were mock-inoculated. A detailed description of plant growth, inoculation and dissection follows. These were applied independently for each paired observation. In order to get potato seedlings, 12 *Solanum tubersum *cv. Desiree explants (stem including an axillary bud) were sectioned under sterile conditions from in vitro growing seedlings and transferred to Magenta boxes containing Gamborg's media. Four weeks post transferring to Gamborg's media, these seedlings were planted, each in a pot containing autoclaved quartz sand and grown under 16 h light per diem at 60% humidity. During seedling growing to plants, eggs of *Meloidogyne javanica *root-knot nematode were extracted from greenhouse cultures, and second-stage juveniles were hatched [38]. Three weeks post transferring the seedlings to pots, 6 plants were inoculated each with 5000 *M. javanica *juveniles, where 6 plants were mock-inoculated with tap, sterile water. Roots from 6 infected and 6 non-infected plants were collected 5 or 10 days post nematode inoculation. Roots were observed under the microscope, and nematode feeding sites were selectively dissected from young lateral roots. To control the effect of tissue sectioning on gene expression, young lateral roots of the non-infected plants were dissected similarly to the infected roots, and collected. Dissected roots were snap-frozen in liquid nitrogen and immediately stored in a -80°C freezer. Consequently, biological material of 8 sources (of 2 time-points × 2 replicates × 2 treatments) was subject to RNA extraction.

### RNA samples

Total RNA was extracted with the RNeasy Kit (Qiagen, Valencia, CA) from infected and non-infected roots to yield 8 test RNA samples. In addition, equal amounts of 20 ug were pooled from all test RNA samples to form a reference sample, in which every test sample was equally represented [39]. The test and reference RNA samples were subjected to amplification with the MessageAmp aRNA kit (Ambion, Austin, TX), using 2.5 to 5 μg of total RNA as starting material. These resulted in 9 samples, a reference sample and 8 test samples designated: '5a-', '5a+', '10a-', '10a+', '5b-', '5b+', '10b-', '10b+' for non-infected (-) or infected (+), 5 or 10 d and first (a) or second (b) biological replicate.

### Microarray experiment design

Each test RNA sample was co-hybridized with the reference RNA sample to both a potato microarray and a tomato microarray, according to a microarray experiment reference design [39]. This meta-design enabled us to compare CSH to SSH in 'paired observations' statistical design; with 8 independent observations (i.e. 8 RNA samples that were "treated" with both CSH and SSH). All heterologous hybridizations to the tomato microarray (i.e., the PT microarray experiment) were performed in HK lab. All homologous hybridizations of the potato RNA to the potato microarray (i.e. the PP microarray experiment) were performed in TIGR labs.

### RNA labeling, microarray hybridizations and data acquisition

For the PT experiment, RNA was labeled so that all the test samples were dyed with Cy5 and the reference sample was dyed with Cy3. A detailed description of the methods used for RNA labeling, microarray hybridization and data acquisition, as in [32].

For the PP experiment, RNA was labeled such that all the test samples were dyed with Cy3 and the reference sample was dyed with Cy5. A detailed description of the methods used for RNA labeling, microarray hybridization and data acquisition can be found in [40].

Results of both experiments are available at GEO [26]; accession no. GSE3584.

### Data normalization

Similar data normalization was performed for both PT and PP data. The output files of the PT and PP experiments were normalized according to GeneSpring (GeneSpring 5.1; Silicon Genetics, Redwood City, CA). First, all PP hybridization results were inverted to gain a test-sample:reference-sample ratios, similar to those of the PT experiment. Then, for each microarray experiment (PP or PT), Lowess normalization [41] was applied. Of the data 35% served for smoothing. In addition, control channel values <40 were set to 40. Lastly, the data was filtered to include only present and marginal calls. The normalization process resulted in 2 expression matrices, one of the PP and the other of the PT experiment.

### Whole chip (transcriptomic) data analysis

Whole chip data analysis was performed separately for PP and for PT in a similar manner. For each experiment the following analysis procedures were performed. To determine the reproducibility of hybridization quality, the reference sample data were exported from GeneSpring to a text file. Excel (Microsoft) was used to determine correlation coefficients between pairs of reference sample data of eight hybridizations. This resulted in 28 comparisons, whose correlation coefficients – their averages and the standard-deviation values – were calculated. Next, GeneSpring was used to perform a Principal Component Analysis (PCA; [42]) on 8 test-sample:reference-sample hybridization data.

For signal reproducibility between the experiments, the span from the lower 0.1 to the upper 99.9 percentiles was determined for each test-samples:reference-sample ratio. These values were compared between PP and PT hybridization data, for each of the 8 RNA sample hybridizations (e.g. between PT '5a+' and PP '5a+' data) by dividing the PT value by that of PP. The average and standard deviation was calculated for these 8 resulted ratios. Next, the (infected-) test-sample:reference-sample ratios were averaged over biological replicates and plotted as transcriptional profiles between 5 d and 10 d. Lastly, differentially regulated genes, with expression ratio of >2 fold or <0.5 fold between infected and non-infected samples (i.e. between the average of '5+' data and that of '5-' data, and between the average of '10+' data and that of '10-' data) were identified by using GeneSpring.

### Construction of tomato-potato microarray matched probe sets

In order to generate tomato-microarray – potato-microarray clone match probe sets, unigene match probe sets were initially generated. For this purpose, the tomato microarray unigene sequences served as queries for a local BLAST search against the potato microarray unigene sequences. Unigene sequences of the tomato and the potato microarrays were retrieved from the manufacturers (CGEP and TIGR, respectively). A database index file was created for the potato microarray unigenes by using the NCBI FORMATDB utility. The tomato microarray unigene sequences served as queries for NCBI local BLASTN against the potato microarray unigene database. The search was limited to E-values <e-01. The BLASTN search gave a list of homologous unigene pairs along with their match information, including E-values and bit-scores.

For each potato-tomato pair of unigenes the associated potato and tomato microarray clones were reconstructed to generate clone matched probe sets. For each potato-tomato unigene pair, each of the tomato unigene associated clones was paired with each of the potato unigene associated clones. This resulted in a list of clone pairs, which formed potato-microarray – tomato-microarray clone matched probe sets. These clone match probe sets were denoted as the unigene-based matched probe sets, and served for the construction of SEM (see below).

### Construction of the System Expression Matrix (SEM)

The unigene-based matched probe sets were used to merge the corresponding data of PP and PT expression matrices (exported from GeneSpring) into one system expression matrix (SEM) using a Matlab (The Mathworks, Natick, MA) script. The merge included for each pair of matched clones the hybridizations data, pair-wise match information (including E-values and bit-scores), and a flag that indicated the number of unigenes associated with a tomato clone (i.e. 1 or 2 for non-chimeric or chimeric clones).

### Analysis of the System Expression Matrix (SEM)

The system expression matrix was uploaded to GeneSpring such that, for each biological replicate, the infected:non-infected data was applied. Additional normalization was done by setting all values below 0.01 to 0.01 and by dividing all values by the median of the corresponding biological replicate values at each time-point examined (e.g. all '5a' infected:non-infected values were divided by their median). The following are analytical steps applied for SEM data. First, Centered Pearson correlation was used for hierarchical clustering of biological replicates for the whole SEM data and for SEM subset data. The latter corresponded to a range of bit-scores (or E-values), in order to find the bit-score (or E-value) threshold for a perfect match between PP and PT (i.e. all biological replicates are clustered over PP and PT). Then, PCA was performed for both SEM whole data, and for sub SEM data that corresponded to the bit-score (or E-value) threshold (disclosed before). Lists of genes, that were differentially regulated (i.e. had >2 fold or <0.5 fold change in expression) between nematode-infected and non-infected plants were extracted for each time-point by filtering on fold change on the averaged biological replicate (i.e. the average of '5a' and '5b', and the average '10a' and '10b'). Corresponding differentially regulated gene lists were intersected (e.g. PP 5 d upregulated gene list was intersected with PT 5d upregulated gene list).

## Abbreviations

CGEP: Center for Gene Expression Profiling

CSH: Cross-Species Hybridization

E-value: Expectation value

GEO: Gene Expression Omnibus

MYA: Million Years Ago

NCBI: National Center for Biotechnology Information

PCA: Principal Component Analysis

SEM: System Expression Matrix

SGN: Solanaceae Genomics Network

SSH: Species-Specific hybridization

TC: TIGR Consensus

TIGR: The Institute for Genomic Research

## Authors' contributions

CB conceived the study, participated in its design, in the extraction and amplification of plant RNA, performed the hybridizations, scanning and quantification of the PT microarray experiment, executed the study analysis and participated in drafting the manuscript and writing the final draft of the manuscript; MB carried out plant growth and inoculation; TG participated in extraction and amplification of plant RNA; YK participated in the design of the study and revision of the manuscript; HC participated in the design of the study and revision of the manuscript; HK supervised the study, participated in its design and led its coordination and the writing of the manuscript. All authors read and approved the final manuscript.

## Supplementary Material

Additional File 1 **Tomato – Potato homologous unigene pairs**. A list of unigene pairs, of tomato unigenes that are represented by the tomato microarray, each paired with its homologous potato unigene. Each pair record contains a tomato SGN unigene ID, a potato TIGR consensus (TC) ID, and the E-value of their match; an Excel file. Click here for file

Additional File 2 **System Expression Matrix (SEM)**. A list of clone pairs of the potato microarray spotted clones, each paired with a tomato microarray spotted clone (be noted that this clone pairing for SEM based on matching the clones associated unigenes). Each pair record contains: a tomato SGN clone ID, a tomato SGN unigene ID, a potato TIGR clone ID, a potato TIGR Concensus (TC) ID, E-value and bit-score of) of the two unigenes homology blast match, the tomato clone-unigene association type (i.e., 2 for chimeric or 1 for non-chimeric), and the infected:non-infected ratios of each of the biological replicate for the PP and the PT experiments; an Excel file. Click here for file

Additional File 3 **Differentially regulated gene list**. (**A**) Lists of differentially regulated genes of each experiment (i.e., PP or PT) for each time point (5 or 10 d) and for each tendency of regulation (up or down).(**B**) Lists of differentially regulated genes that were found mutual between PP and PT., for each time point (5 or 10 d) and for each tendency of regulation (up or down). Differentially regulated genes were detected for a subset of the SEM data, which corresponded to bit score ≥ 129 (E value ≤ e-26). Each gene record includes tomato SGN unigene ID, potato TIGR Consensus (TC) ID, fold change of gene expression (including average and range over the biological replicates); an Excel file. Click here for file

Additional File 4 **Differentially regulated gene list**. Lists of differentially regulated genes that were found mutual between PP and PT., for each time point (5 or 10 d) and for each tendency of regulation (up or down). Differentially regulated genes were detected for a subset of the SEM data, which corresponded to bit score ≥ 129 (E value ≤ e-26). Each gene record includes tomato SGN unigene ID, potato TIGR Consensus (TC) ID, fold change of gene expression (including average and range over the biological replicates); an Excel file. Click here for file
